# Unifying Diagnosis Identification and Prediction Method Embedding the Disease Ontology Structure From Electronic Medical Records

**DOI:** 10.3389/fpubh.2021.793801

**Published:** 2022-01-20

**Authors:** Jingfeng Chen, Chonghui Guo, Menglin Lu, Suying Ding

**Affiliations:** ^1^Health Management Center, The First Affiliated Hospital of Zhengzhou University, Zhengzhou, China; ^2^School of Economics and Management, Institute of Systems Engineering, Dalian University of Technology, Dalian, China

**Keywords:** unifying diagnosis, disease ontology structure, set similarity measure, clustering, electronic medical records

## Abstract

**Objective:**

The reasonable classification of a large number of distinct diagnosis codes can clarify patient diagnostic information and help clinicians to improve their ability to assign and target treatment for primary diseases. Our objective is to identify and predict a unifying diagnosis (UD) from electronic medical records (EMRs).

**Methods:**

We screened 4,418 sepsis patients from a public MIMIC-III database and extracted their diagnostic information for UD identification, their demographic information, laboratory examination information, chief complaint, and history of present illness information for UD prediction. We proposed a data-driven UD identification and prediction method (UDIPM) embedding the disease ontology structure. First, we designed a set similarity measure method embedding the disease ontology structure to generate a patient similarity matrix. Second, we applied affinity propagation clustering to divide patients into different clusters, and extracted a typical diagnosis code co-occurrence pattern from each cluster. Furthermore, we identified a UD by fusing visual analysis and a conditional co-occurrence matrix. Finally, we trained five classifiers in combination with feature fusion and feature selection method to unify the diagnosis prediction.

**Results:**

The experimental results on a public electronic medical record dataset showed that the UDIPM could extracted a typical diagnosis code co-occurrence pattern effectively, identified and predicted a UD based on patients' diagnostic and admission information, and outperformed other fusion methods overall.

**Conclusions:**

The accurate identification and prediction of the UD from a large number of distinct diagnosis codes and multi-source heterogeneous patient admission information in EMRs can provide a data-driven approach to assist better coding integration of diagnosis.

## Introduction

In medical practice, clinicians are encouraged to seek a unifying diagnosis (UD) that could explain all the patient's signs and symptoms in preference to providing several explanations for the distress being presented (1). A UD is a critical pathway to identify the correct illness and craft a treatment plan; thus, clinical experience and knowledge play an important role in the science of diagnostic reasoning. Generally, from a brief medical history from a patient, clinicians can use the intuitive system in their brain and rapidly reason the disease types, whereas for complex and multi-type abnormal results, clinicians must use the more deliberate and time-consuming method of analytic reasoning to deduce the UD, raising the risk of diagnostic errors (2).

To increase the accuracy of a UD, enhancing individual clinicians' diagnostic reasoning skills and improving health care systems are regarded as two important approaches to support clinicians through the diagnostic process. The former requires professional knowledge training and lifelong learning, whereas the latter mainly involves the development of information technology (3). For an individual clinician, an intelligent clinical decision support system is prone to acceptable and can help clinicians to improve their unifying diagnostic decisions (4). Recently, along with the widespread adoption of electronic medical records (EMRs), an extremely large volume of electronic clinical data has been generated and accumulated (5, 6). Meanwhile, artificial intelligence and big data analytic technology have been successfully applied to clinical diagnostic procedures and treatment regimen recommendation, which has resulted in new opportunities for intelligent clinical decision support systems that use data-driven knowledge discovery methods (7–10).

From the data mining perspective, a UD aims to classify a large number of distinct diagnosis codes reasonably according to the disease taxonomy and attempt to adopt a disease to summarize or explain various clinical manifestations of the disease. Therefore, the nature of a UD is diagnosis code assignment along with disease correlation exploitation. Diagnosis code assignment refers to the clinical decision process in which supervised methods are adopted to predict and annotate disease codes based on patients' medical history, signs and symptoms, and laboratory examination (11). According to the number of diagnosis codes that patients suffer from, diagnosis code assignment can be divided into single-label (12), multi-class (13), multi-label (14), and multi-task learning methods (15). However, although many novel supervised learning models have been proposed and can achieve high performance in terms of assigning diagnosis codes for new patients using frontier supervised methods, such as ensemble learning (16), reinforcement learning (17), and deep learning (18), they cannot further explore disease co-occurrence relations for UD identification and prediction.

The coexistence of multiple diseases is pervasive in the clinical environment, particularly for patients in the intensive care unit (ICU) (19). According to the statistical results of the MIMIC-III database, which is a freely accessible critical care database, the average number of diagnosis codes for patients in the ICU is 11. Additionally, diagnosis codes are highly fine-grained, closely related, and extremely diverse (20). For example, the patient with admission identifier (ID) 100223 is assigned to 28 ICD-9 codes, and many diagnosis codes are similar, such as 276.2 (Acidosis, order: 15), 276.0 (Hyperosmolality and/or hypernatremia, order: 18), and 276.6 (Hyperpotassemia, order: 26). Thus, it is trivial and difficult for clinicians to make a consistent, accurate, concise, and unambiguous diagnostic decision reasonably.

Furthermore, although the inter-relation of diagnosis codes was considered in previous studies, the researchers commonly used the first three digits of ICD-9 codes to assign diagnosis codes for patients (21–23); hence, the complexity may increase and prediction performance may reduce when considering all digits of the ICD-9 codes. Additionally, in those studies, reasonable complicated and confused diagnosis codes could not be classified into a UD using a data-driven method. A UD is the basic principle of clinical diagnostic thinking. Its basic idea is that when a patient has many symptoms, if these symptoms can be explained by one disease, it will never explain different symptoms using multiple diseases (1). A UD reflects the integrity of the patient and the professionalism of clinicians; however, in previous studies, the main focus was on the UD of a category of diseases from the clinical perspective, such as mood/mental disorders (24), intracranial mesenchymal tumor (25), and arrhythmogenic right ventricular cardiomyopathy (26). In this study, we fully consider the fine-grained diagnosis codes (i.e., all digits) of patients, identify the UD from a group of patient diagnostic information using an unsupervised clustering method and predict the UD for new unseen patients using multi-class learning methods.

## Materials and Methods

### Data Collection

**Figure 1:**
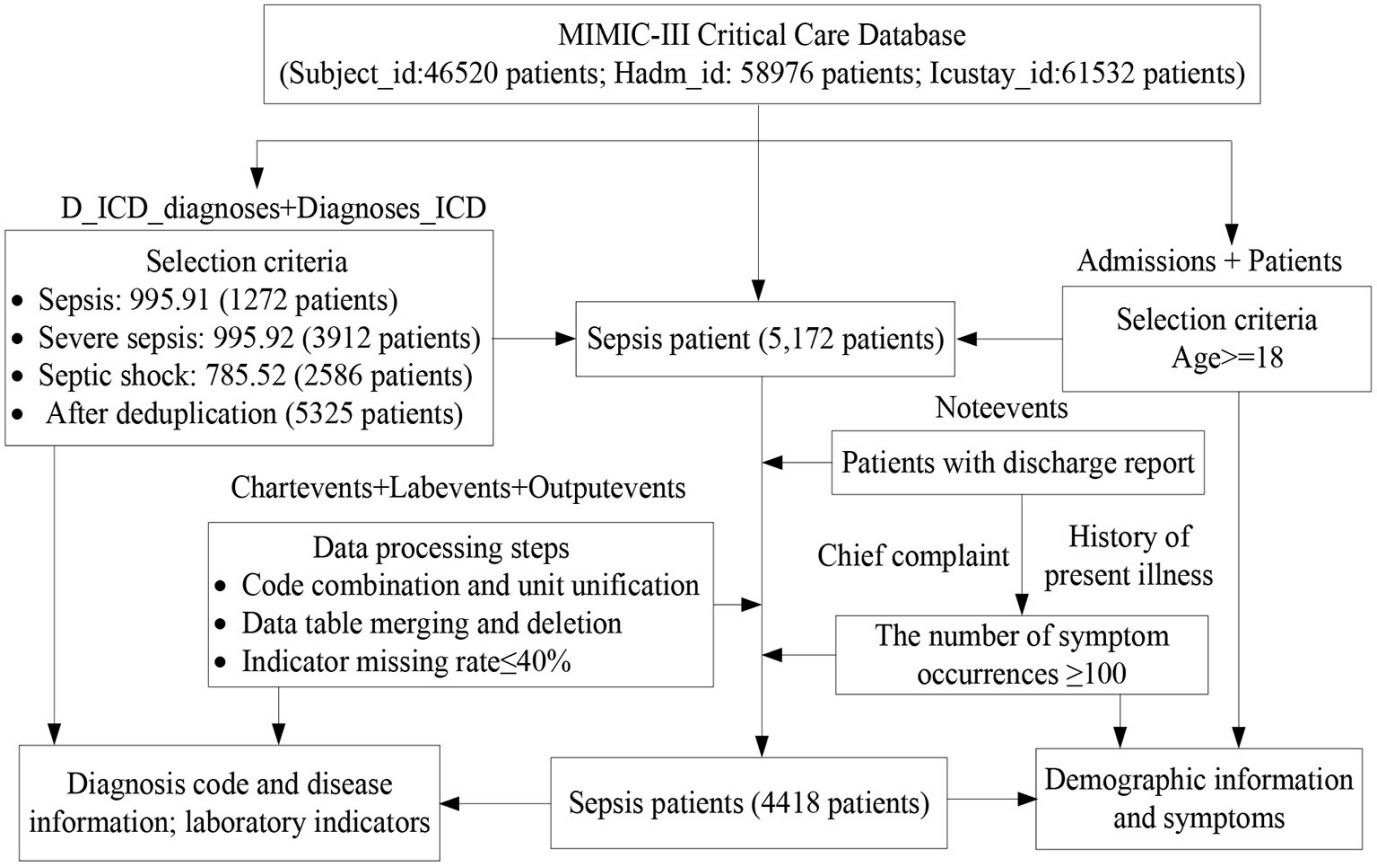
Dataset selection of sepsis patients from the MIMIC-III database.

We selected a dataset of sepsis patients from the MIMIC-III database, where sepsis is divided into general sepsis, severe sepsis, and septic shock (27, 28). Figure 1 shows the detailed processes of data collection and preprocessing of sepsis patients, including the identification of sepsis patients, data extraction, data cleaning, and feature selection. Finally, we screened 4,418 sepsis patients and extracted their diagnostic information to unify the diagnosis identification, their demographic information, laboratory examination information, chief complaint, and history of present illness information, and obtain a UD prediction.

First, the diagnostic information of 4,418 sepsis patients mainly contained the patient hospital admission ID (Hadm-id), ICD-9 diagnosis code, order of diagnosis code, and a brief definition of the diagnosis codes, where the sum, maximum, minimum, and average numbers of diagnosis codes were 80501, 39, 3, and 18.3, respectively. Additionally, for the visualization, we removed duplicate diagnosis codes and converted the remaining 3,070 diagnosis codes into digital numbers from 1 to 3,070. The Supplementary Table 1 shows the diagnostic information of two patients.

Then, for the health condition of patients admitted to hospital, we used the minimum, maximum, median, mean, and variance value as the 5-tuple features of each laboratory indicator, and designed a symptom identification method based on text analysis of patient discharge reports, including rule setting, text segmentation, text extraction, abbreviation dictionary construction, negative word recognition, case unification, word segmentation, stop word removal, and external symptom dictionary embedding (Supplementary Figure 1). Additionally, we added related indicators to measure patients' severity, such as AIDS, hematologic malignancy, metastatic cancer SOFA, SAPS, and SAPS-II. Finally, we obtained 120 features of the health condition of sepsis patients in the experimental dataset, as shown in Table 1.

**Table 1:** Feature information of the health condition of sepsis patients.

**Information**	**Feature**	**Description (Range, Type)**
Demographic information	Admission type	Emergency, elective, urgent (Nominal)
	Gender	Female, male (Nominal)
	Age	[18, 89] (Numeric)
Laboratory examination information	Potassium Level, PO2, serum bicarbonate level, temperature, sodium level, urine out foley, urea nitrogen, WBC, bilirubin level, GCSmotor, GCSeyes, HR, GCSverbal, NBP, RR, SPO2, hemoglobin, platelet count, creatimine	Minimum, maximum, median, mean, and variance value (Numeric)
Symptom information	Fever, abdominal pain, shortness of breath, nausea and vomiting, weakness, diarrhea, dizziness, palpitation, cough, fatigue, discomfort, dysuria, shock, weight change, loss of appetite, and night sweating	0, 1 (Nominal)
Related indicators	AIDS, hematologic malignancy, metastatic cancer	0, 1 (Nominal)
	SOFA, SAPS, and SAPS-II	Integer (Numeric)

### Method

**Figure 2:**
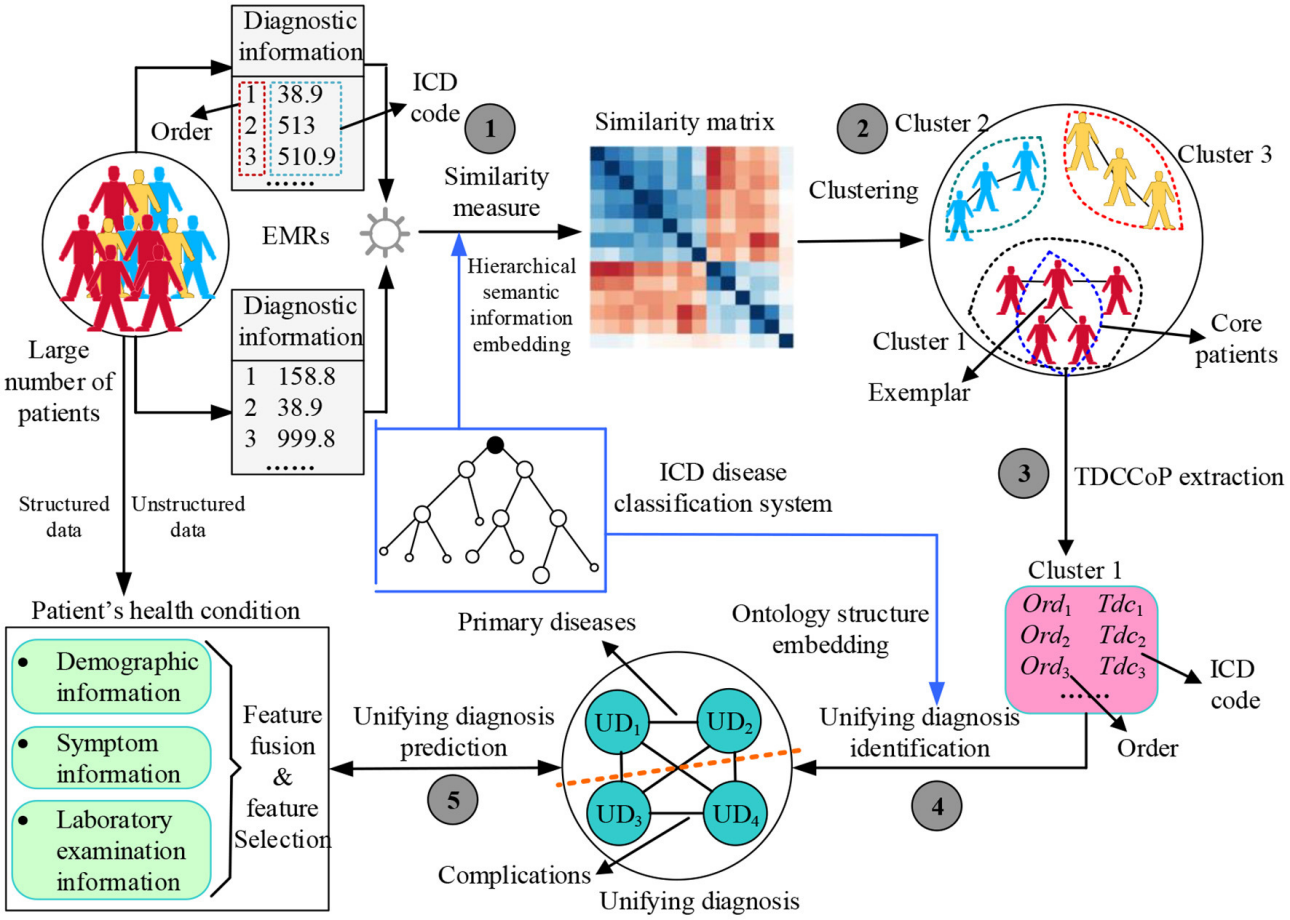
Research framework for applying the proposed UDIPM to EMRs.

Figure 2 shows the proposed UD identification and prediction method (UDIPM), which uses four types of information from EMRs. We adopt diagnostic information to identify the UD, and use demographic information, symptom information, and laboratory examination information to predict the UD. First, we apply a set of similarity measure methods to a large number of patients by embedding the semantic relation of the ICD classification system (Task 1 in Figure 2). Second, we apply a clustering algorithm to the similarity matrix to divide patients into different groups, and further obtain the exemplar and core patients of each cluster (Task 2 in Figure 2). Third, we extract the typical diagnosis code co-occurrence patterns (TDCCoP) from each cluster by defining a threshold and a sorting function (Task 3 in Figure 2). Fourth, we combine the visual analysis and conditional co-occurrence matrix (CCoM) to identify the UD by selecting the optimal segmentation (Task 4 in Figure 2). Finally, after obtaining the health condition of the patient admitted to hospital, we obtain a UD prediction using multi-class classification methods (Task 5 in Figure 2).

### Patient Similarity Measure Method

Many methods exist for measuring patient similarity (29, 30). In this study, considering the semantic relations of diagnosis codes in the ICD ontology structure, we adopt a set similarity measure method. First, we define patient diagnostic information as a series of ordered diagnosis codes. Then we reconstruct the ontology structure based on a disease classification system to easily measure patient similarity. Finally, we describe the process of the set similarity method, including the information content (IC) measure of diagnosis codes, diagnosis code similarity measure, and diagnosis code set similarity measure.

#### Patient's Diagnostic Information Representation

Diagnostic information refers to a record of disease diagnosis made by clinicians based on the health condition of a patient admitted to hospital. It is stored in the patient's EMR data in the form of a diagnosis code (e.g., ICD-9 and ICD-10). Because of the prevalence of disease complications, a patient's EMR is typically annotated using multiple disease codes, and these codes have a certain priority (i.e., order). The higher the priority of the diagnosis code is, the more central and important the disease is for this patient, then the weaker conversely. Thus, patient diagnostic information can be represented as

(1) $$\begin{array}{r} D=\left\{\left(dc_{1},Ord\left(dc_{1}\right)\right),\left(dc_{2},Ord\left(dc_{2}\right)\right),\cdots \;,\left(dc_{i},Ord\left(dc_{i}\right)\right),\cdots \;\right\}, \end{array}$$

where *dc*_*i*_ and *Ord*(*dc*_*i*_) represent the *i*-th diagnosis code and its order, respectively.

#### Ontology Structure Construction

**Figure 3:**
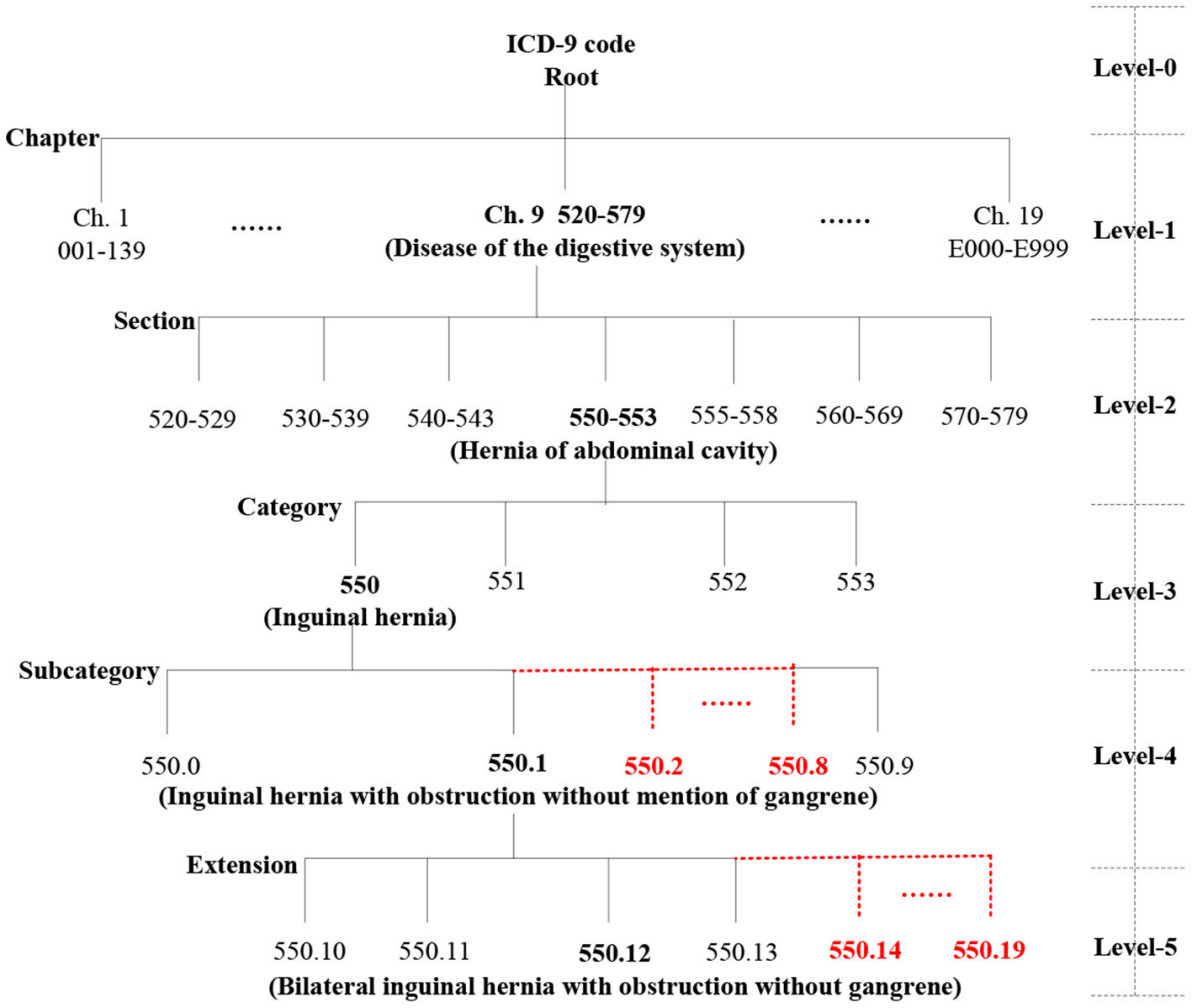
Local ontology structure of ICD-9 codes.

We automatically construct a five-level ICD-9 ontology structure, shown in Figure 3, in which level-0 is the virtual root node, level-1 has 19 chapters, level-2 has 129 sections, level-3 has ~1,300 categories (Supplementary Figure 2), and the last two levels are expanded to 10 types of sub-nodes under each node. For example, level-4 contains 550.0, 550.1, 550.2 (virtual code), 550.3 (virtual code), … and 550.9, and level-5 includes 550.10, 550.11, 550.12, 550.13, 550.14 (virtual code), … 550.19 (virtual code). More importantly, the actual diagnosis codes of patients belong to the ICD-9 ontology structure, whereas the virtual codes are only used to construct a complete ICD ontology structure and do not play a role in the actual similarity measure.

#### Set Similarity Measure

##### Information Content Measure of Diagnosis Codes

In the ICD-9 ontology structure, each code represents a concept, and there is semantic similarity between classification concepts. Additionally, concepts on the same branch are more similar than those on different branches. Thus, we use the level depth measure method of the hierarchical tree (29), that is, we assign a value to each level of the ICD-9 ontology structure; the deeper the concept level, the larger the value. For an ICD-9 code *dc*_*i*_, the IC is defined as

(2) $$\begin{array}{r} IC\left(dc_{i}\right)=level\left(dc_{i}\to Root\right), \end{array}$$

where *Root* is the virtual root node and the function *level*(.) denotes the level depth from the ICD-9 code *d*_*i*_ to the root node. Intuitively, the IC of the root node (level-0) is 0, the ICs of a chapter (level-1), section (level-2), category (level-3), subcategory (level-4), and extension (level-5) are 1, 2, 3, 4, and 5, respectively.

##### Code-Level Similarity Measure

For the IC of codes, there are several approaches to measure code-level similarity. We use the least common ancestor (LCA) of two codes to measure the similarity of diagnosis codes, defined as

(3) $$\begin{array}{r} s\left(dc_{i},dc_{j}\right)=\frac{2IC\left(LCA\left(dc_{i},dc_{j}\right)\right)}{IC\left(dc_{i}\right)+IC\left(dc_{j}\right)}, \end{array}$$

where *dc*_*i*_ and *dc*_*j*_ are two diagnosis codes, and *LCA*(*dc*_*i*_, *dc*_*j*_) is the LCA of *dc*_*i*_ and *dc*_*j*_. If *dc*_*i*_ = *dc*_*j*_, then *LCA*(*dc*_*i*_, *dc*_*j*_) = *dc*_*i*_ = *dc*_*j*_, and *IC*[*LCA*(*dc*_*i*_, *dc*_*j*_)] = *IC* (*dc*_*i*_) = *IC* (*dc*_*j*_). If *dc*_*i*_ ≠ *dc*_*j*_ and *LCA*(*dc*_*i*_, *dc*_*j*_) = *Root*, then *IC*[*LCA*(*dc*_*i*_, *dc*_*j*_)] = 0.

**Figure 4:**
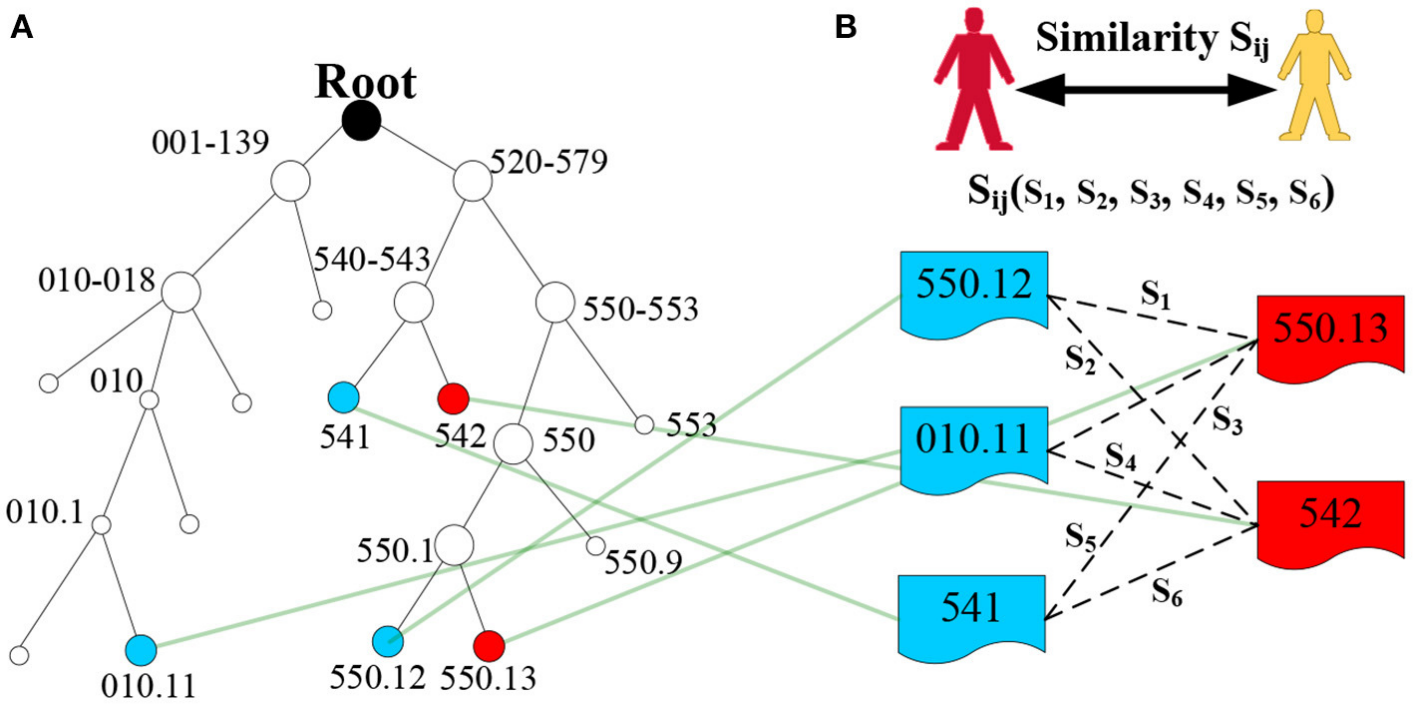
Example of LCA generation in the ICD-9 ontology structure. **(A)** Denotes the ICD-9 ontology structure, and **(B)** denotes the diagnosis codes of two patients.

To make this concept easier to understand, we provide a simple example in Figure 4A. Thus, *LCA*(550.12, 550.13) = 550.1, *LCA*(541, 550.13) = 520–579, *s* = *s*_1_(550.12, 550.13) = 2*IC*(550.1)/[*IC*(550.12) + *IC*(550.13)] = 2 ^*^ 4/(5 + 5) = 0.8.

##### Code Set-Level Similarity Measure

In the EMR dataset, patient diagnostic information is typically a set of diagnosis codes. Thus, patient similarity can be transformed into the similarity of the diagnosis code set. Generally, for binary code-level similarity, we can use classical methods, such as Dice, Jaccard, cosine, and overlap, to calculate set-level similarity. However, these methods cannot fully embed semantic similarity. Thus, we use the most similar concept pair's average value to measure the set-level similarity (29), and the formula is defined as

(4) $$\begin{array}{l} S\left({D^{\prime }}_{i},{D^{\prime }}_{j}\right)=\\ 1-\frac{\left(\underset{dc_{ig}\in {D^{\prime }}_{i}}{\sum }\min_{dc_{jh}\in {D^{\prime }}_{j}}\left(1-s\left(dc_{ig},dc_{jh}\right)\right)+\underset{dc_{jh}\in {D^{\prime }}_{j}}{\sum }\min_{dc_{ig}\in {D^{\prime }}_{i}}\left(1-s\left(dc_{jh},dc_{ig}\right)\right)\right)}{\left|{D^{\prime }}_{i}|+|{D^{\prime }}_{j}\right|}, \end{array}$$

where $D_{i}^{\prime }$ and $D_{j}^{\prime }$ are the diagnostic information of patient *i* and patient *j*, respectively, which does not consider the order of diagnosis codes; that is, $D_{i}^{\prime }$={*dc_i_*_1_, *dc_i_*_2_,…, *dc_i_*_g_,…} and $D_{j}^{\prime }$={*dc_j_*_1_, *dc_j_*_2_,…, *dc*_*jh*_,…}. |$D_{i}^{\prime }$| and |$D_{j}^{\prime }$| are the number of diagnosis codes for patient *i* and patient *j*, and *dc*_*ig*_ and *dc*_*jh*_ are the *g*-th diagnosis code of patient *i* and the *h*-th diagnosis code of patient *j*, respectively. Finally, we obtain the similarity **S**_*ij*_ of the two patients (Figure 4B), and similarity matrix **S** for all patients in the EMRs using a set similarity measure method. The pseudocode of the patient similarity measure method is presented in Algorithm 1.

**Algorithm 1:**
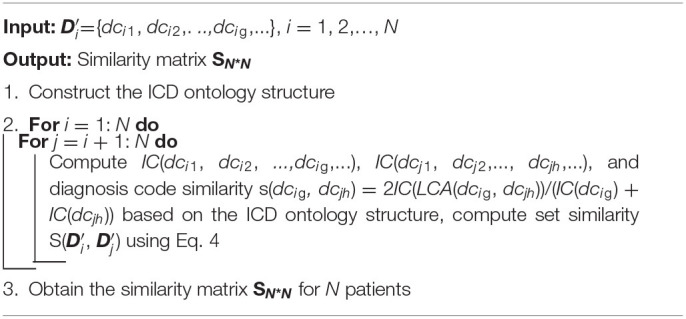
Patient similarity measure method.

### Patient Clustering Algorithm

A clustering algorithm aims to divide patients into multiple groups based on the similarity matrix **S**, requiring that patients in the same group are as similar as possible, and patients in different groups are as dissimilarity as possible (31, 32). In this study, considering the advantages, such as not predefining the number of clusters, the real existence of exemplars, and much lower error, we adopt affinity propagation (AP) clustering (33, 34).

AP clustering determines the number of clusters by controlling the input exemplar preferences (*p*), where *p* is more robust than *K* because *p* monotonically controls the perception granularity. Generally, *p* depends on the similarity matrix **S**_***N*****∗*****N***_, number of input patients (*N*), and *p* coefficient (*p*_*coe*_), which is represented as

(5) $$\begin{array}{r} p=median\left(\text{S}\right)-p_{coe}*N. \end{array}$$

After patients are clustered, we identify *K* clusters (***C***_1_, ***C***_2_,…, ***C***_*K*_), and define the popularity (i.e., support) of each cluster as

(6) $$\begin{array}{r} Support\left(C_{k}\right)=\frac{\sum_{j\in \left\{1,2,\cdots \;,N\right\}}\lambda \left(C\left({D^{\prime }}_{j}\right),E\left(C_{k}\right)\right)}{N},k=1,2,\cdots \;,K, \end{array}$$

where ***C***($D_{j}^{\prime }$) represents the cluster to which patient *j* belongs and *E*(***C***_*k*_) denotes the exemplar of ***C***_*k*_. λ(.) is an indicator function; if patient *j* belongs to ***C***_*k*_, then λ[***C***($D_{j}^{\prime }$), *E*(***C***_*k*_)] = 1; otherwise, λ[***C***($D_{j}^{\prime }$), *E*(***C***_*k*_)] = 0.

Additionally, we obtain the sum of similarities (SS), which is an important indicator used to evaluate clustering performance. The SS depends on the similarity matrix **S**_N*N_, number of input patients (*N*), number of clusters (*K*), and corresponding exemplars, which is represented as

(7) $$\begin{array}{r} SS\left(K\right)=\sum_{i=1}^{K}\sum_{{D^{\prime }}_{j}\in C_{i}}S\left({D^{\prime }}_{j},E\left(C_{i}\right)\right). \end{array}$$

Generally, the larger the SS value, the better the clustering performance. The pseudocode of the patient clustering algorithm is presented in Algorithm 2.

**Algorithm 2:**
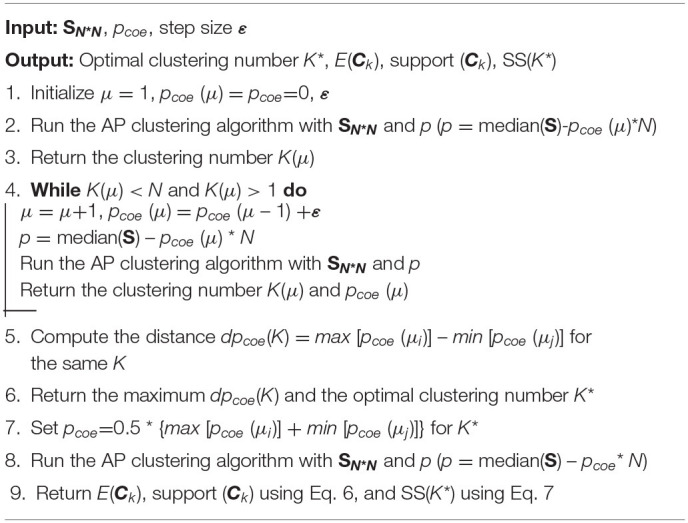
Patient clustering algorithm.

### TDCCoP Extraction Method

In our previous studies, we proved that defining the core zone of a cluster is an effective approach to extract stable clustering results (35). Additionally, considering the complex semantic relations among different diagnosis codes, the feature of a cluster cannot be fully described when the diagnostic information (cluster center or exemplar) of only one patient is used. Thus, we also define the core zone of each cluster to select a group of patients (i.e., core patients) using the *k*-nearest neighbor method, and further extract typical diagnosis codes (TDCs). For cluster ***C***_*k*_, the core zone is defined as

(8) $$\begin{array}{r} Core_{k}=\left\{{D^{\prime }}_{j}|S\left({D^{\prime }}_{j},E\left(C_{k}\right)\right)\geq \tau \right\}, \end{array}$$

where *E*(***C***_*k*_) is the exemplar of cluster ***C***_*k*_ and τ is a similarity threshold defined in advance, which aims to determine the number of core patients.

Then, for cluster ***C***_*k*_, the occurrence probability of the diagnosis code *dc*_*h*_ can be represented as

(9) $$\begin{array}{r} Prob_{k}\left(dc_{h}\right)=\frac{\sum_{{D^{\prime }}_{j}\in Core_{k}}\lambda \left(dc_{h}\text{,}{D^{\prime }}_{j}\right)}{\left|Core_{k}\right|},h=1,\cdots \;,H, \end{array}$$

where |Core_*k*_| denotes the number of core patients in cluster ***C***_*k*_. λ(.) is an indicator function; if the diagnostic information $D_{j}^{\prime }$ of patient *j* contains diagnosis code *dc*_*h*_, then λ (*dc*_*h*_, $D_{j}^{\prime }$) = 1; otherwise, λ (*dc*_*h*_, $D_{j}^{\prime }$) = 0. *H* is the number of all diagnosis codes after duplicates are deleted.

After we calculate the probability of all diagnosis codes in the cluster ***C***_*k*_, we define the TDC as

(10) $$\begin{array}{r} Tdc_{h}=\left\{dc_{h}|Prob_{k}\left(dc_{h}\right)>\delta_{1}\right\}, \end{array}$$

where δ_1_ is a threshold defined in advance to differentiate high-frequency and low-frequency diagnosis codes.

Based on all TDCs of the cluster ***C***_*k*_, we further analyze the priority of TDCs by embedding the order of the patient diagnostic information, that is, for patient *j, D*_*j*_= {[*dc_j_*_1_, *Ord*(*dc_j_*_1_)], [*dc_j_*_2_, *Ord*(*dc_j_*_2_)], [*dc*_*jh*_, *Ord*(*dc*_*jh*_)], …} and *D*_*j*_′ = {*dc_j_*_1_, *dcj*_2_, *dc*_*jh*_, …}. Thus, the average order (AOrd) of TDC *Tdc*_*h*_ is defined as

(11) $$\begin{array}{l} AOrd\left(Tdc_{h}\right)=\frac{\sum_{{D^{\prime }}_{j}\in Core_{k},Tdc_{h}\in {D^{\prime }}_{j}}Ord_{D_{j}}\left(Tdc_{h}\right)\lambda \left(Tdc_{h}\text{,}{D^{\prime }}_{j}\right)}{\sum_{{D^{\prime }}_{j}\in Core_{k},Tdc_{h}\in {D^{\prime }}_{j}}\lambda \left(Tdc_{h}\text{,}{D^{\prime }}_{j}\right)},\\ \;h=1,\cdots \;,H^{\prime }, \end{array}$$

where *H*′ is the number of TDCs in cluster ***C***_*k*_ and *Ord*_*Dj*_(*Tdc*_*h*_) denotes the order of TDC *Tdc*_*h*_ in the diagnostic information *D*_*j*_ of patient *j*. Generally, the smaller the AOrds of typical diagnostic codes, the more likely they are to be primary diseases.

Finally, after obtaining TDCs and their AOrds, we define a sorting function to determine TDCCoP, which is represented as

(12) $$\begin{array}{l} TDCCoP_{k}=Sort\left(\left(Tdc_{1},AOrd_{k}\left(Tdc_{1}\right)\right),\cdots \;,\left(Tdc_{H^{\prime }},AOrd_{k}\left(Tdc_{H^{\prime }}\right)\right)\right)\\ \;=\left\{\left(Tdc_{1},Ord^{\prime }\left(Tdc_{1}\right)\right),\cdots \;,\left(Tdc_{H^{\prime }},Ord^{\prime }\left(Tdc_{H^{\prime }}\right)\right)\right\}, \end{array}$$

where *Ord*′ (*Tdc*_*h*_) is the new order of *Tdc*_*h*_. For example, if cluster ***C***_*k*_ has only three TDCs (e.g., *Tdc*_1_, *Tdc*_2_, and *Tdc*_3_) and its AOrds are 5.3, 7.8, and 3.8, respectively, then after sorting, the *TDCCoP*_*k*_ is {(*Tdc*_3_, 1), (*Tdc*_1_, 2), (*Tdc*_2_, 3)}. The pseudocode of the TDCCoP extraction method is presented in Algorithm 3.

**Algorithm 3:**
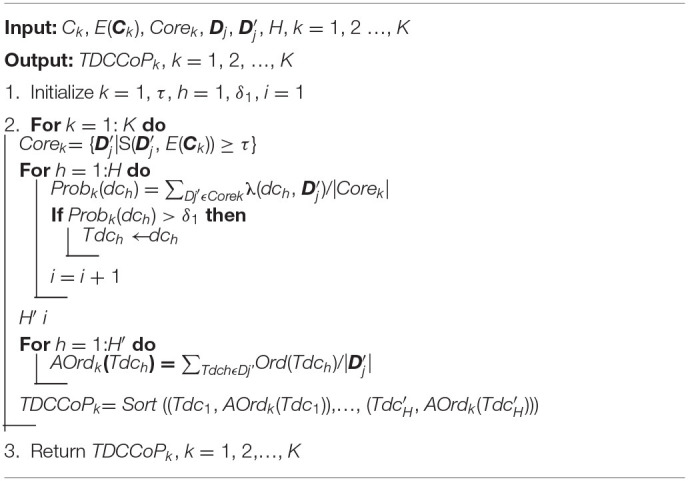
TDCCoP extraction method.

### UD Identification Method

**Figure 5:**
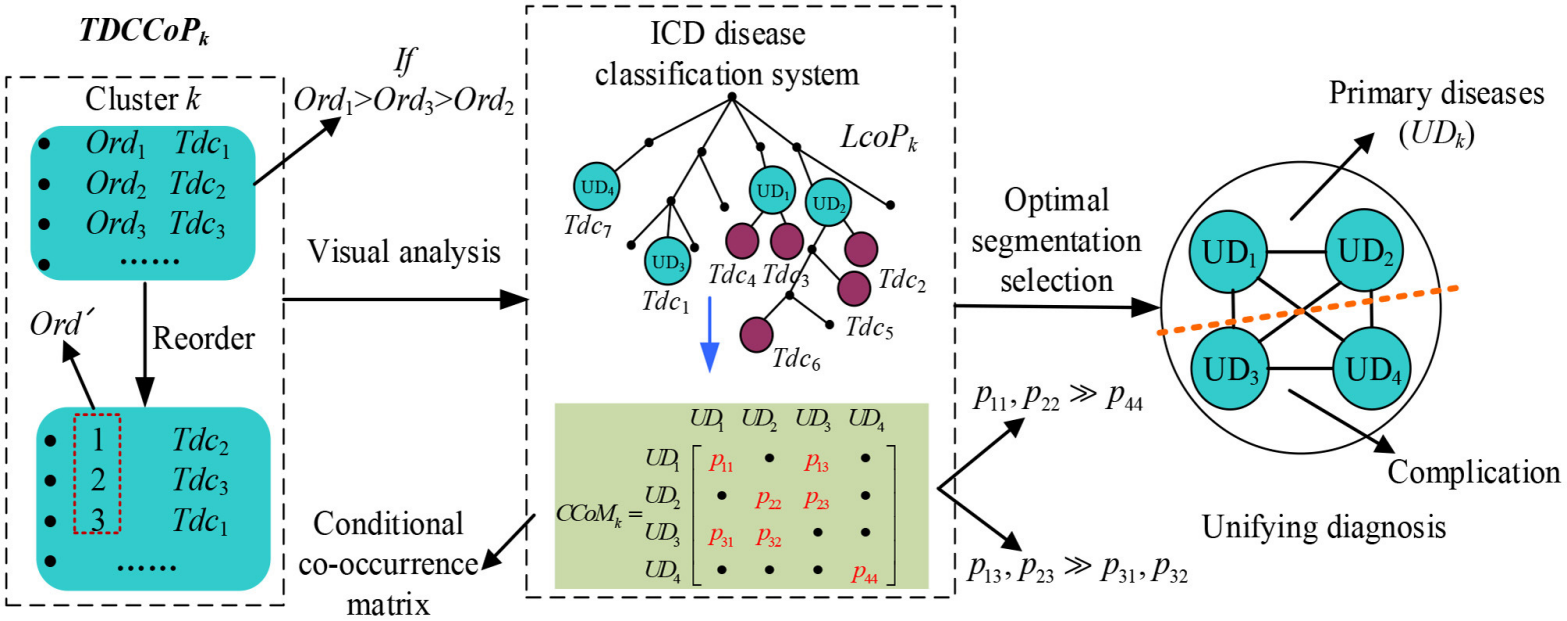
Proposed UD identification method.

To identify a UD, categorizing the TDCCoP of each cluster reasonably according to the disease taxonomy is a critical step. In this study, we propose a UD identification method, as shown in Figure 5. Specifically, for the *TDCCoP*_*k*_ of cluster *k*, we first visualize all TDCs in the reconstructed ICD ontology structure, and mark their orders. Then we use the LCA method to categorize these codes, and define their LCA and the corresponding orders. Furthermore, we calculate the CCoM using patient diagnostic information to select the optimal segmentation between primary diseases and complications. Finally, we regard the identified primary diseases as the UD.

First, we define the LCA co-occurrence pattern (LCoP) of the *TDCCoP*_*k*_ using visual analysis of the ICD ontology structure as

(13) $$\begin{array}{l} LCoP_{k}=\{d_{i}|d_{i}=LCA_{\left\{Tdc_{1},Tdc_{2},\cdots \;\right\}\in TDCCoP_{k}}\left(Tdc_{1},Tdc_{2},\cdots \;\right),\\ \;d_{i}\neq Root\}. \end{array}$$

Then we calculate the order of each *d*_*i*_ in *LCoP*_*k*_ as

(14) $$\begin{array}{l} Ord(d_{i}\text{)=}min_{d_{i}=LCA\left(Tdc_{1},Tdc_{2},\cdots \;,Tdc_{m}\right)}\\ \;\left(Ord^{\prime }\left(Tdc_{1}\right),Ord^{\prime }\left(Tdc_{2}\right),\cdots \;,Ord^{\prime }\left(Tdc_{m}\right)\right), \end{array}$$

where *m* is the number of TDCs in *LCoP*_*k*_ whose LCA is *d*_*i*_.

Additionally, considering the causal relation between *d*_*i*_ and *d*_*j*_ in *LCoP*_*k*_, we define the conditional co-occurrence probabilities *p*_*k*_(*d*_*j*_/*d*_*i*_) and *p*_*k*_(*d*_*i*_/*d*_*j*_) as

(15) $$\begin{array}{r} \begin{array}{c} p_{k}\left(d_{j}/d_{i}\right)=Freq_{k}\left(d_{j},d_{i}\right)/Freq_{k}\left(d_{i}\right)\\ p_{k}\left(d_{i}/d_{j}\right)=Freq_{k}\left(d_{i},d_{j}\right)/Freq_{k}\left(d_{j}\right) \end{array}, \end{array}$$

where *Freq*_*k*_ (*d*_*i*_, *d*_*j*_) and *Freq*_*k*_ (*d*_*j*_, *d*_*i*_) denote the number of co-occurrences of *d*_*i*_ and *d*_*j*_, respectively, and *Freq*_*k*_(*d*_*i*_) denotes the number of occurrences of *d*_*i*_ in the cluster ***C***_*k*_.

Thus, for all diagnosis codes in *LCoP*_*k*_, we generate a CCoM C**CoM**_*k*_, where C**CoM**_*k*_ (*i, j*) = *p*_*k*_(*d*_*j*_/*d*_*i*_), **CoM**_*k*_ (*j, i*) = *p*_*k*_(*d*_*i*_/*d*_*j*_), and the diagonal entry C**CoM**_*k*_ (*i, i*) = *p*_*k*_(*d*_*i*_) = *Freq*_*k*_(*d*_*i*_)/|Core_*k*_|. If C**CoM**_*k*_ (*i, j*) >> C**CoM**_*k*_ (*j, i*) or C**CoM**_*k*_ (*i, i*) >> C**CoM**_*k*_ (*j, j*) exist, then *d*_*j*_ is more prone to occur after the occurrence of *d*_*i*_; thus, *d*_*i*_ is more likely to be a primary disease, whereas *d*_*j*_ will become a complication, and vice versa.

After analyzing the precedence relation of all diagnosis codes in *LCoP*_*k*_ using C**CoM**_*k*_, we obtain the optimal segmentation between primary diseases and complications, and define the UD of cluster *k* as

(16) $$\begin{array}{r} UD_{k}=\left\{d_{i}|d_{i}\in LCoP_{k},d_{i}\neq Complication\right\}, \end{array}$$

where ***UD***_*k*_ is a set of primary diseases. The pseudocode of the UD identification method is presented in Algorithm 4.

**Algorithm 4:**
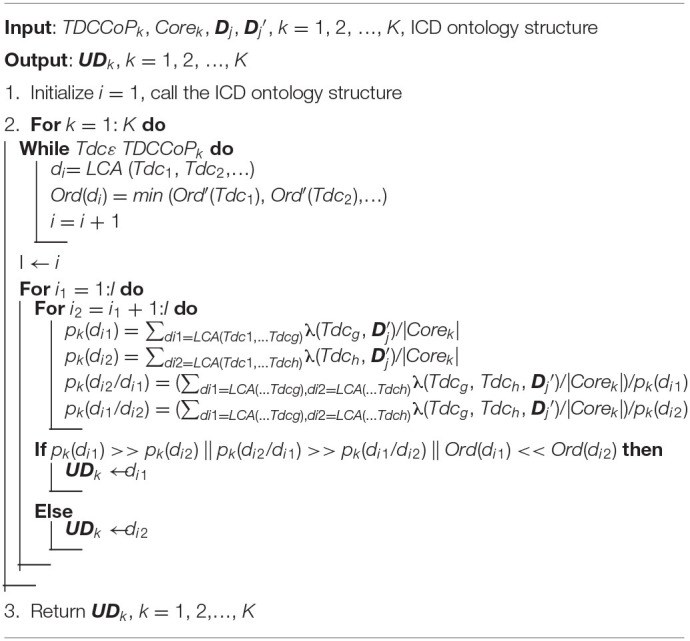
UD identification method.

### UD Prediction Method

**Figure 6:**
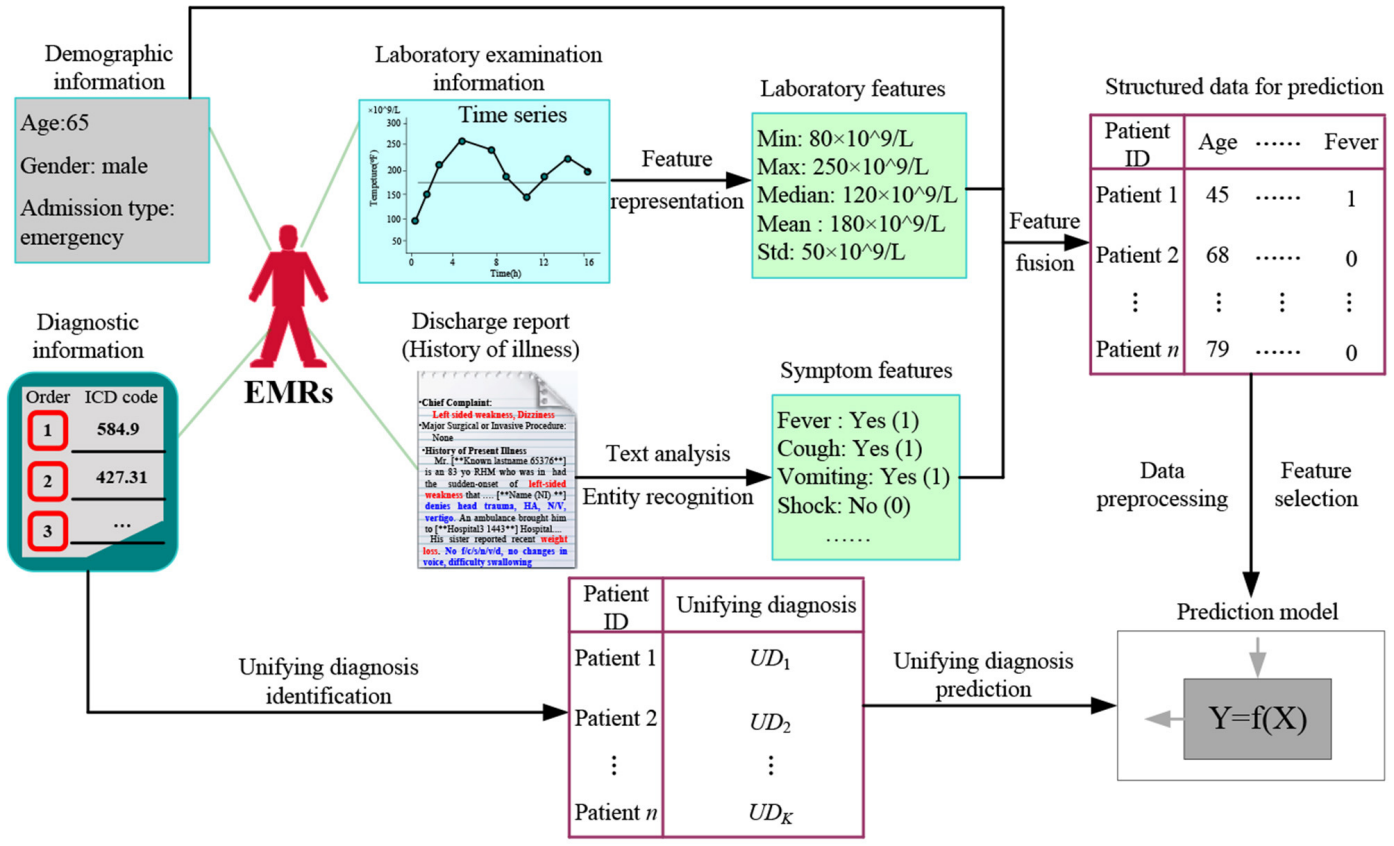
Proposed UD prediction method.

After identifying the UD, we further study the prediction task based on the health condition of a patient admitted to hospital, exploring the important features to assign the most possible UDs to new patients. Figure 6 shows the proposed UD prediction method. First, we extract three categories of features using time series feature representation and text analysis methods, and fuse them in structured data for further prediction. Then after data pre-processing and feature selection, we label all patients with a UD. Finally, we adopt classical prediction models to perform the UD prediction task.

#### Patient's Health Condition Representation

The health condition of a patient admitted to hospital includes demographic information, symptom information, and laboratory examination information, which play crucial roles for clinicians in diagnosing disease types, evaluating disease severity, and designing a treatment regimen.

##### Demographic Information

Demographic information mainly includes the date of birth, age, gender, admission type, marital status, occupation, and residence, defined as

(17) $$\begin{array}{r} De=\left\{De^{Age},De^{Gender},De^{Admission\;Type},De^{M\text{arital }Status},\cdots \right\}. \end{array}$$

##### Symptom Information

Symptom information is recorded in the chief complaint and history of present illness in the form of text, where the chief complaint is the most painful part of the disease process, including the main symptoms and onset time. The history of present illness describes the entire process for the patient after suffering from diseases, including occurrence, development, evolution, diagnosis, and treatment. Thus, the patient's symptom information can be represented as.

(18) $$\begin{array}{r} Sy=\left\{Sy^{Fever},Sy^{Weakness},Sy^{Diarrhea},\cdots \right\}. \end{array}$$

##### Laboratory Examination Information

Laboratory examination refers to an indirect judgment of the health condition as a result of measuring specific components of blood and body fluids using instruments. Laboratory indicators typically have the characteristics of a time series, particularly for patients in the ICU. Thus, we use the minimum value, maximum value, median value, mean value, and variance of laboratory indicators to represent the time series, defined as

(19) $$\begin{array}{r} LE=\{\{(min\left(LE^{WBC}\right),max\left(LE^{WBC}\right),med\left(LE^{WBC}\right),\\ mean\left(LE^{WBC}\right),var\left(LE^{WBC}\right)\},\cdots \;\} \end{array}$$

Finally, we obtain the health condition of a patient admitted to hospital using a feature fusion method, that is, ***X*** = {***De***; ***Sy***; ***LE***}.

#### Information Gain-Based Feature Selection

Before predicting the UD, to remove noisy data, reduce the complexity and dimensionality of the dataset, and achieve accurate results, it is essential to apply feature selection methods to identify useful features. Therefore, feature selection is an important step that improves the clarity of the data and decreases the training time of prediction models (4). In this study, we use the information gain (IG) method to measure the importance of features and eliminate some irrelevant features. Then we compute the IG of feature *x*_*i*_ as

(20) $$\begin{array}{l} IG\left(x_{i}\right)=H\left(Y\right)-H\left(Y/x_{i}\right)\\ \;=-\overset{K}{\underset{k=1}{\sum }}P\left(y_{k}\right)\log P\left(y_{k}\right)+\overset{K}{\underset{k=1}{\sum }}P\left(y_{k}/x_{i}\right)\log P\left(y_{k}/x_{i}\right), \end{array}$$

where feature *x*_*i*_ϵ***X***, ***Y*** = {***UD***_1_,…, ***UD***_*k*_,…, ***UD***_*K*_}, *y*_*k*_ϵ***Y***, *H*(***Y***), and *H*(***Y***/*x*_*i*_) denote the information entropy and conditional information entropy given feature *x*_*i*_ for a UD classification, and *P*(*y*_*k*_) and *P*(*y*_*k*_/*x*) denote the probability of *y*_*k*_ and condition probability of *y*_*k*_ given feature *x*_*i*_, respectively.

Thus, we obtain the important features as

(21) $$\begin{array}{r} X^{\prime }=\left\{x_{i}|IG\left(x_{i}\right)>\delta_{2}\right\}, \end{array}$$

where δ_2_ is a threshold defined in advance to differentiate the important and unimportant features using the IG method.

#### Prediction Model Establishment

After obtaining the feature representation and UD result of each patient, we generate a standard dataset (*Y* and *X*′) and establish a prediction model [*Y* = *f* (*X*′)]. In this study, we apply five classifiers to achieve a UD prediction: logistic regression, decision tree, random forest, SVM, and extreme gradient boosting (XGBoost). In the prediction process, we adopt the *Z*-fold cross-validation (CV) method, which randomly partitions the initial dataset into *Z* mutually exclusive subsets, and perform training and testing *Z* times. We set *Z* to 5 or 10. Then we compute the average CV error to determine the prediction model as

(22) $$\begin{array}{r} CVError_{Z}=\frac{1}{Z}\overset{Z}{\underset{z=1}{\sum }}L_{z}\text{=}\frac{1}{Z}\overset{Z}{\underset{z=1}{\sum }}\frac{1}{m_{z}}\overset{m_{z}}{\underset{j=1}{\sum }}{\left({ŷ}_{j}-y_{j}\right)}^{2}, \end{array}$$

where *L*_*z*_ and *m*_*z*_ are the average CV error and number of the *z*-th testing dataset, and *y*_*j*_ and ŷ_*j*_ are the real and predicted UDs of the *j*-th patient, respectively.

Additionally, we identify distinctive features of different unifying diagnoses by analyzing the feature importance ranking results.

### Parameter Setting

In our experiment, we set 5 parameters in advance. First, we set *p*_*coe*_ in Eq. 5 to select the number of clusters, and then τ in Eq. 8, which is a similarity threshold to determine the number of core patients (i.e., |Core|). We discuss both parameters based on the stability of the experimental results. We set δ_1_ in Eq. 10 to 0.3 to obtain TDCs, and δ_2_ in Eq. 21 to 0.005 to select the important features. We set the last parameter *Z* in Eq. 22 to 10 to perform the 10-fold CV method. In particular, before UD prediction, we used data pre-processing methods, that is, data normalization and smoothing for imbalanced classes.

## Results

### Selection of the Cluster Number

**Figure 7:**
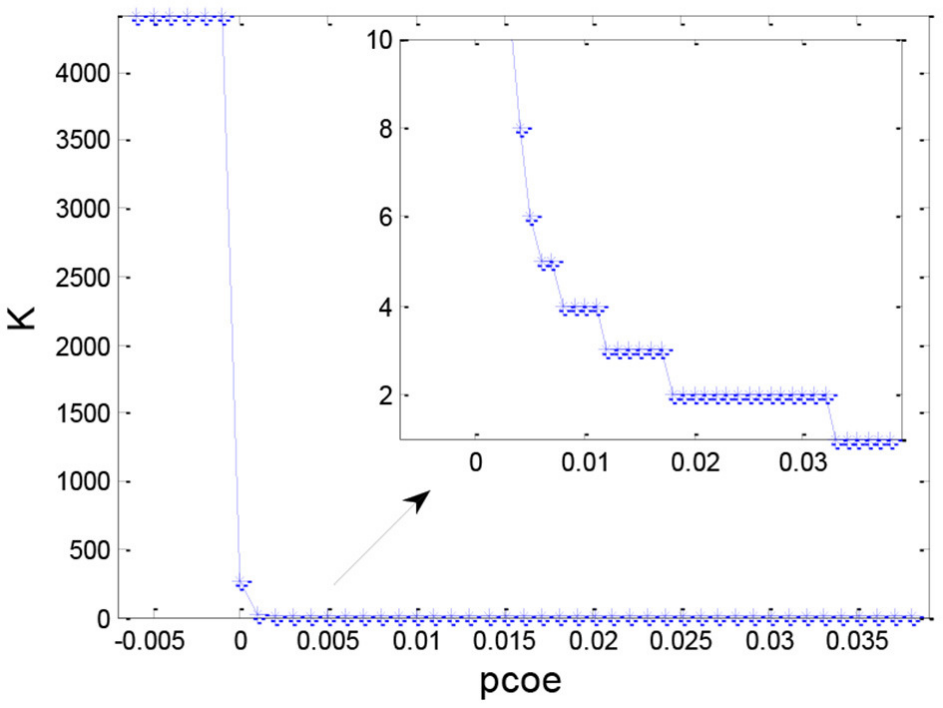
Distribution of the number of clusters for different values of *p*_*c*_.

After obtaining the set similarity measure based on the ontology structure for 4,418 sepsis patients, we obtained the similarity matrix **S** and used the AP clustering algorithm to divide all the patients into multiple groups. Figure 7 shows the distribution of the number of clusters under different values of *p*_*c*_. Generally, the number of clusters decreased as the preference coefficient increased. The most stable number of clusters was two when *p*_*c*_ ranged from 0.018 to 0.032. Thus, we selected two clusters (*p*_*c*_ = 0.025) to identify TDCs and extract TDCCoPs from each cluster.

### Stability Analysis of TDCs

**Figure 8:**
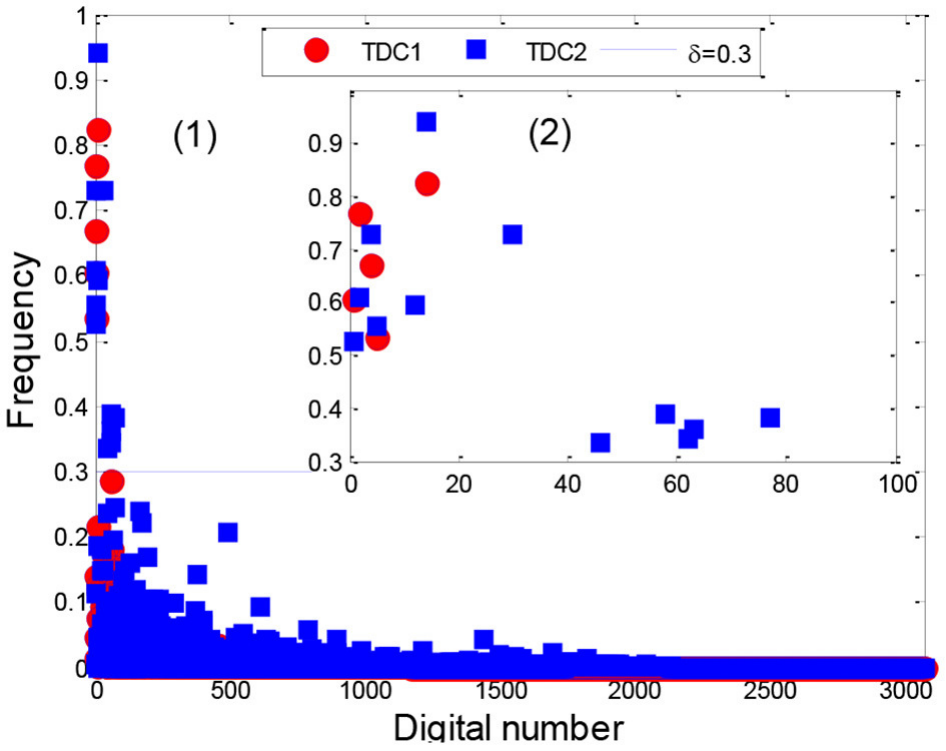
Distribution of TDCs for 800 core patients.

After applying the AP clustering algorithm, we first divided the 4,418 sepsis patients into two clusters, where cluster 1 and 2 contained 1,391 and 3,027 patients with a support of 31.48% and 68.52%, respectively. Then we analyzed the stability of the TDCs in Eq. 10 using a set of different numbers of core patients in Eq. 8 (|Core|=100, 200, 400, 500, 800, and all patients), as shown in Figure 8, Supplementary Figure 3.

From the distribution of TDCs in Figure 8, Supplementary Figure 3, the results showed that the stable range of core patients was from 400 to 800 (five codes in cluster 1 and 12 codes in cluster 2) because the number of TDCs and their distributions were approximately coincident. Specifically, compared with the stable TDCs, more TDCs were identified when the number of core patients was set to 100 and 200 (14 codes in cluster 2), such as the digital number 71 (276, disorders of fluid electrolyte and acid-base balance) and digital number 490 [V58.610, long-term (current) use of anticoagulants] (Supplementary Figures 3A,B). Digital number 99 (995.91, sepsis) was identified in cluster 1, and another three codes (486, 276.2, and 250) were not identified in cluster 2 (Supplementary Figure 3E) when we used all patients in the two clusters to extract TDCs. Thus, in the next experiment, we set the number of core patients to 800 to extract the TDCCoPs.

### TDCCoP Extraction From Each Cluster

**Figure 9:**
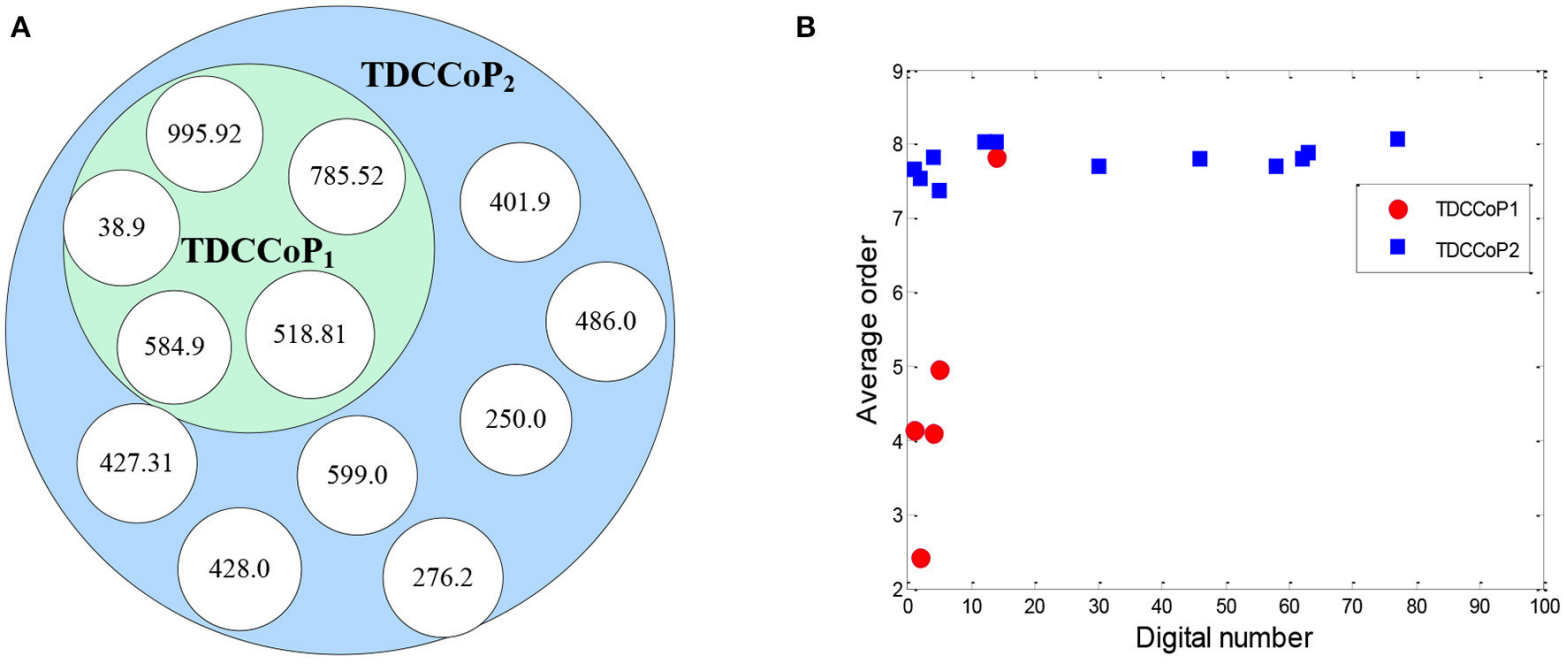
Co-occurrence relation and AOrd of all TDCs. **(A)** Co-occurrence relation. **(B)** AOrd.

Using the clustering results, we finally determined two clusters, selected 800 core patients from each cluster, and set δ to 0.3 in Eq. 10 to identify TDCs and extract TDCCoPs. Figure 9 shows the co-occurrence relation and AOrd of all TDCs in two TDCCoPs, and Table 2 provides a detailed description of all TDCs in the two TDCCoPs.

**Table 2:** Detailed description of three *TDC*s.

**TDCCOP**	**Digital** **number**	**TDC**	**Definition of** **diagnosis code**	**Occurrence** **frequency**	**Average** **order**	**Re-order**
TDCCOP_1_	1	518.81	Acute respiratory failure	0.604	4.145	3
(1391)	2	38.9	Septicemia	0.769	2.411	1
	4	785.52	Septic shock	0.669	4.090	2
	5	584.9	Acute kidney failure	0.534	4.956	4
	14	995.92	Severe sepsis	0.824	7.816	5
TDCCOP_2_	1	518.81	Acute respiratory failure	0.526	7.665	3
(3027)	2	38.9	Septicemia	0.608	7.545	2
	4	785.52	Septic shock	0.729	7.813	8
	5	584.9	Acute kidney failure	0.554	7.377	1
	12	427.31	Atrial fibrillation	0.593	8.038	11
	14	995.92	Severe sepsis	0.941	8.031	10
	30	428.0	Congestive heart failure	0.729	7.703	5
	46	486.0	Pneumonia organism	0.334	7.805	6
	58	599.0	Urinary tract infection	0.389	7.701	4
	62	401.9	Essential hypertension	0.343	7.807	7
	63	276.2	Acidosis	0.360	7.875	9
	77	250.0	Diabetes mellitus without complication	0.383	8.062	12

To summarize, the experimental results indicated that there were 12 types of TDCs in the two TDCCoPs, where TDCCoP_1_ and TDCCoP_2_ had 5 and 12 codes, respectively. Specifically, the two TDCCoPs had similarities and differences. There were three similarities: (1) Five types of TDCs were the same, that is, 518.81, 38.9, 785.52, 584.9, and 995.92. (2) The AOrds of all TDCs in the same TDCCoPs were similar, for example, the AOrds of four TDCs in TDCCoP_1_ were all below 6, whereas those of the TDCs in TDCCoP_2_ were over 7. (3) The TDCs 38.9 (septicemia), 785.52 (septic shock), and 995.92 (severe sepsis) had the highest occurrence probability in the two TDCCoPs. There were also three differences: (1) TDCCoP_2_ identified more TDCs than TDCCoP_1_. (2) The occurrence probabilities of TDCs in TDCCoP_1_ were larger than those in TDCCoP_2_. (3) The AOrds of the same TDC were different in the two TDCCoPs, for example, 518.81 (acute respiratory failure) in the two TDCCoPs was 4.145 and 7.665, respectively. Additionally, septicemia (38.9) was a high-frequency and primary disease in sepsis patients, which is a life-threatening complication that can occur when bacteria from another infection enters the blood and spreads throughout the body.

Furthermore, using Eq. 12 and Algorithm 3, we extracted the TDCCOPs of the two clusters described in Table 2, that is, TDCCOP_1_ = {(38.9, 1), (785.52, 2), (518.81, 3), (584.9, 4), (995.92, 5)} and TDCCOP_2_ = {(584.9, 1), (38.9, 2), (518.81, 3), (599.0, 4), (428.0, 5), (486.0, 6), (401.9, 7), (785.52, 8), (276.2, 9), (995.92, 10), (427.31, 11), (250.0, 12)}. Thus, from a reordering perspective, acute kidney failure, septicemia, and acute respiratory failure were probably the primary diseases in the two TDCCOPs.

### UD Identification Based on TDCCOPs

**Figure 10:**
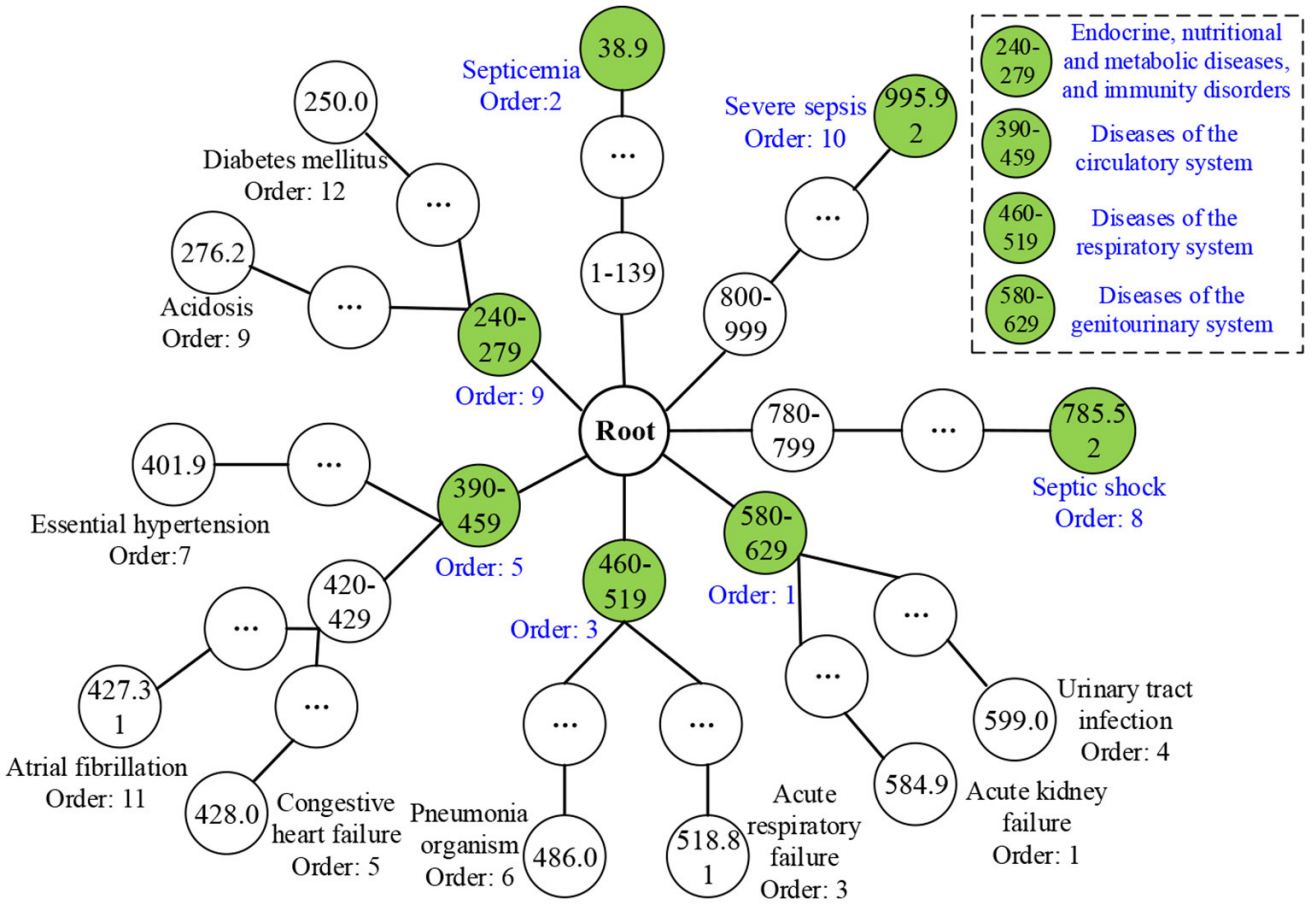
LCoP_2_ identified using the visualization of *TDCoP*_3_ in the ontology structure.

After obtaining TDCCoPs, we visualized all the TDCs in the ICD-9 ontology structure. First, we categorized them using the LCA method to identify LCoPs using Eq. 13. Consider *TDCCoP*_2_ as an example. The visualization result is shown in Figure 10. Clearly, we identified LCoP_2_ with seven types of diseases, which are light green color, and computed the order of the new diseases using Eqs 13, 14: diseases of the genitourinary system (580–629, order: 1), septicemia (38.9, order: 2), diseases of the respiratory system(460–519, order: 3), diseases of the circulatory system (390–459, order: 5), septic shock (785.52, order: 8), endocrine, nutritional, and metabolic diseases, and immunity disorders (240–279, order: 9), and severe sepsis (995.92, order: 10).

Then we calculated the CCoM_2_ of the LCoP_2_ based on the diagnostic information of 800 core patients in cluster 2, as described in Table 3. First, the conditional probabilities *p*({390–459, 995.92}/{580–629, 38.9, 460–519}) colored red were significantly larger than the values *p*({580–629, 38.9, 460–519}/{390–459, 995.92}) colored blue, which indicates that diseases of the genitourinary system (580–629, order: 1), septicemia (38.9, order: 2), and diseases of the respiratory system (460–519, order: 3) were more likely to be primary diseases, whereas diseases of the circulatory system (390–459, order: 5) and severe sepsis (995.92, order: 10) were probably complications.c Second, the orders of septic shock (785.52, order: 8) and endocrine, nutritional, and metabolic diseases, and immunity disorders (240–279, order: 9) were also larger than those of the first three diseases. Thus, diseases of the respiratory system (460–519, order: 3) and diseases of the circulatory system (390–459, order: 5) were likely to be the optimal segmentation between primary diseases and complications, and the first three diseases were considered to be the UD (UD_2_) of cluster 2.

**Table 3:**
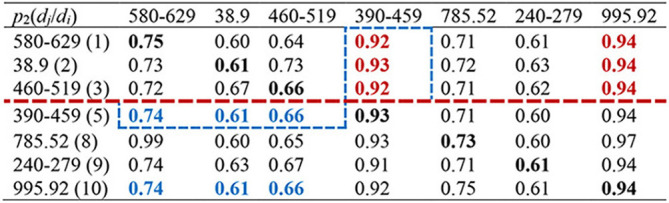
CCoM_2_ of the LCoP_2_.

*Values in brackets are the orders of the seven diseases, bold values on the master diagonal denote the occurrence probabilities of the seven diseases, and values in red and blue are conditional probabilities for distinguishing between primary diseases and complications*.

### UD Prediction Based on Patient Admission Information

**Figure 11:**
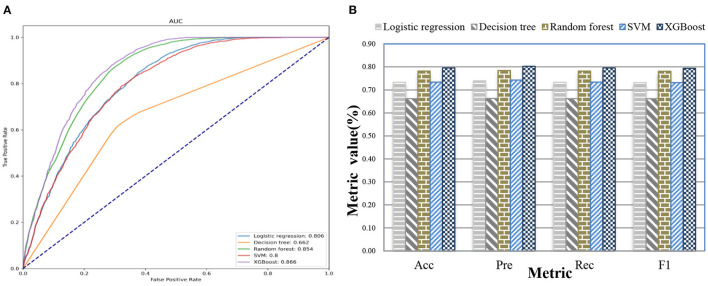
Classification performance of the proposed UDIPM. **(A)** AUC. **(B)** Acc, Pre, Rec, and F1.

**Figure 12:**
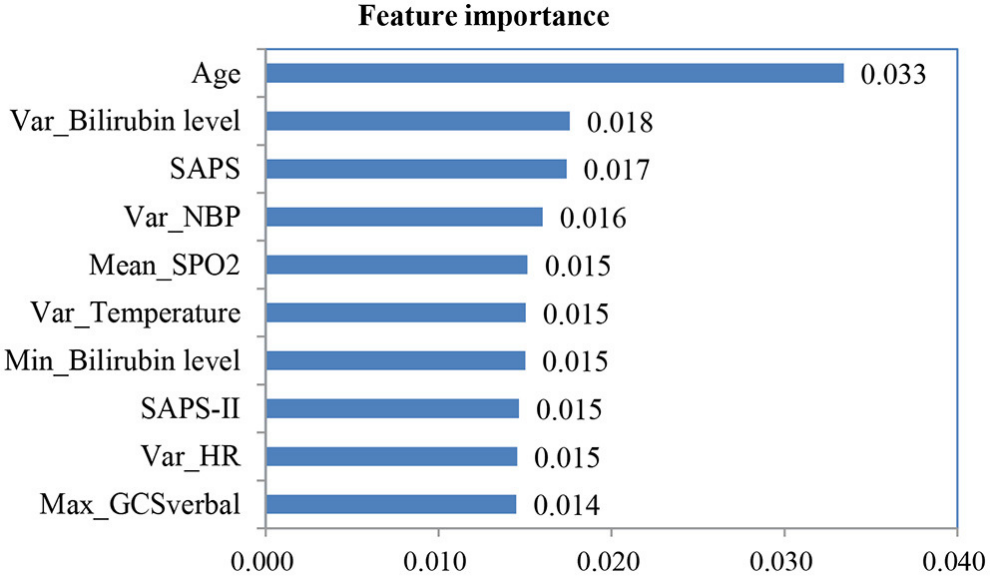
Ten most important features using the random forest method.

After we applied feature fusion and feature selection using the IG method, we further performed five classifications to predict a UD based on patient admission information and identify important features for the constructed prediction models. Figure 11 shows the classification performance of the proposed UDIPM, including the area under the ROC curve (AUC), accuracy (Acc), precision (Pre), recall (Rec), and F1-score (F1), and Figure 12 presents the 10 most important features identified using the random forest method (Supplementary Figure 4).

The experimental results indicated that the proposed UDIPM achieved better prediction performance, where the AUC values were all above 0.8, except for the decision tree method. Similarly, the best Acc, Pre, Rec, and, F1 among all classifications was XGBoost, at ~80%, followed by random forest, SVM, and logistic regression, whereas the decision tree was last, at ~66%. Consider the random forest as an example. We obtained the feature importance results to better understand the prediction model. First, we found that demographic information (i.e., age) and laboratory examination information were more important than symptom information. Then some disease severity indicators were very important, such as SAPS and SAPS-II. Finally, the variance distribution (i.e., Var) of the laboratory examination indicators was more important than the mean, median, minimum, and maximum values. To summarize, the proposed UDIPM not only identified a UD from patient diagnostic information but also predicted a UD based on the health condition of a patient admitted to hospital.

## Discussion

In this study, we conducted various experiments to demonstrate the efficiency of the proposed UDIPM when compared with other methods. Specifically, the proposed UDIPM fused three methods: a set similarity measure method, clustering, and classification algorithms. For the set similarity measure method, we selected Dice, Jaccard, cosine, and overlap as comparative methods, and used *SS* in Eq. 7 as a performance metric based on the AP clustering results. For the classification algorithms, we selected logistic regression, decision tree, random forest, SVM, and XGBoost. Additionally, we used AUC, Acc, Pre, Rec, and F1 as performance metrics to measure the effectiveness of the classification algorithms. The evaluation methods and metrics are described in detail in Table 4.

**Table 4:** Evaluation methods and metrics used in our experiment.

**Method name**	**Set similarity measure**	**Clustering**	**Classification**
The proposed method (UDIPM)	Set similarity based on ontology	AP clustering	Logistic regression
Fusion method 1 (FM1)	Dice = 2|A⋂B|/|*A*| + |*B*|		Decision tree
Fusion method 2 (FM2)	Jaccrd = |A⋂B|/|A⋃B|		Random forest
Fusion method 3 (FM3)	Cosine = $|\text{A}⋂\text{B}|\text{/}\sqrt{\left|A|▪|B\right|}$		SVM
Fusion method 4 (FM4)	Overlap = |A⋂B|/*min* { |*A*|, |*B*| }		XGBoost
			AUC
			Acc = (TP + TN)/N
Metric	*SS* (Eq. 7)		Pre = TP/(TP + FP)
			Rec = TP/(TP + FN)
			F1 = 2Pre*Rec/(Pre + Rec)

*A and B are the diagnosis code sets of two patients, the Dice method is the same as the proposed UDIPM when we do not consider the disease ontology structure and replace the code similarity with $s=\left(dc_{i},dc_{j}\right)\;=\;\{\begin{array}{cc} 1, & ifdc_{i}\;=dc_{j}\\ 0 & otherwise \end{array}$, true positive (TP) and true negative (TN) measure the ability of classifier models to predict the UD, false positive (FP) and false negative (FN) identify the number of false predictions generated by the models, and we used FM to determine the prediction performance*.

**Figure 13:**
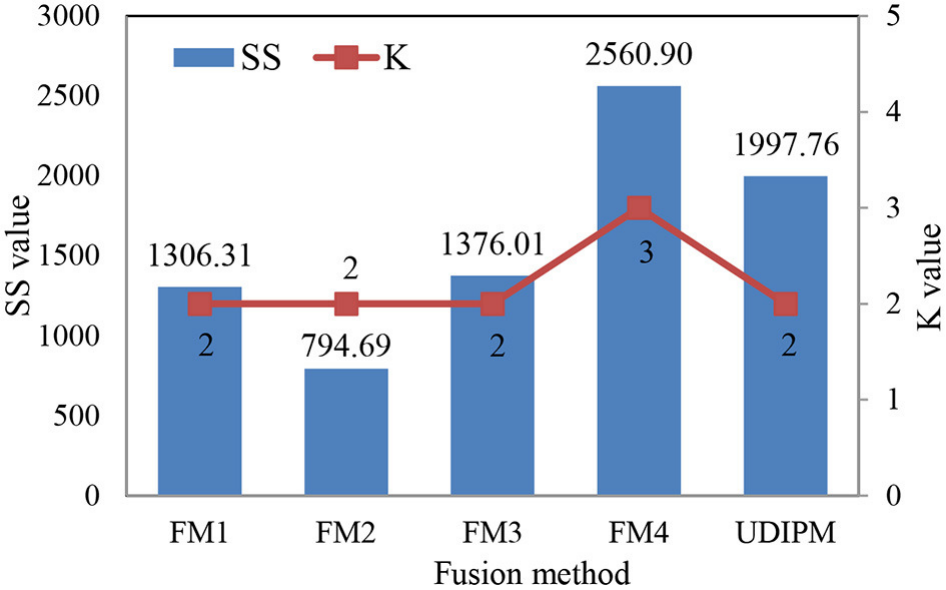
Similarity measure and clustering results of different fusion methods.

The detailed experimental results are shown in Figure 13, Table 5. Specifically, for the set similarity measure, we first selected the optimal number of clusters using AP clustering algorithms, and then computed the SS value based on the clustering results (Algorithm 2). The experimental results indicated that the optimal numbers of clusters for four FMs were 2, 2, 2, and 3 (Supplementary Figure 5), and the proposed UDIPM achieved the second-highest SS value of 1997.86; it was only below FM4 (Figure 13). The reason is that the SS value increased as the cluster number increased. Interestingly, although the similarities of FM1 and FM2 were different, they had the same clustering results.

**Table 5:** Classification results of different fusion methods.

**Fusion** **method**	**Classification** **algorithm**	**Metric**				
		**Acc**	**Pre**	**Rec**	**F1**	**AUC**
FM1	Logistic regression	0.725	0.739	0.725	0.721	0.782
(Dice)	Decision tree	0.682	0.683	0.682	0.682	0.682
FM2	Random forest	0.779	0.782	0.779	0.778	0.851
(Jaccard)	SVM	0.722	0.763	0.722	0.711	0.778
	XGBoost	0.804	0.818	0.804	0.802	0.860
FM3	Logistic regression	0.734	0.743	0.734	0.732	0.804
(Cosine)	Decision tree	0.682	0.683	0.682	0.682	0.682
	Random forest	0.786	0.790	0.786	0.785	0.859
	SVM	0.736	0.752	0.736	0.732	0.801
	XGBoost	0.813	0.821	0.813	0.812	0.884
FM4	Logistic regression	0.465	0.437	0.421	0.411	0.628
(Overlap)	Decision tree	0.388	0.370	0.371	0.369	0.529
	Random forest	0.467	0.434	0.400	0.371	0.620
	SVM	0.471	0.384	0.404	0.350	0.626
	XGBoost	0.481	0.451	0.423	0.404	0.629
UDIPM	Logistic regression	0.733	0.740	0.733	0.732	0.806
	Decision tree	0.662	0.663	0.662	0.662	0.662
	Random forest	**0.782**	**0.784**	**0.782**	**0.781**	**0.854**
	SVM	0.734	0.743	0.734	0.732	0.800
	XGBoost	**0.795**	**0.803**	**0.795**	**0.794**	**0.866**

*Bold values denote the first and second-highest performance using the UDIPM*.

For the classification results obtained using the 10-fold CV method in Table 5, the proposed method achieved the second-highest performance using logistic regression, random forest, and SVM, and the third-highest performance using the decision tree and XGBoost. More importantly, all metrics of the proposed UDIPM were higher than those of FM4. Therefore, from the overall performance evaluation in combination with the set similarity measure, clustering, and classification, the UDIPM was an effective method for identifying and predicting a UD from EMRs.

Further, for all fusion methods, the results of performance comparison indicated that both XGBoost and random forest were superior to other classification algorithms in terms of the Acc, Pre, Rec, F1, and AUC. The main reason is that XGBoost and random forest are ensemble learning algorithms by combining multiple classifiers, which can often achieve more significant generalization performance than a single classifier. Specifically, XGBoost is an improved algorithm based on the gradient boosting decision tree, which can efficiently construct boosted trees and run in parallel. XGBoost works by combining a set of weaker machine learning algorithms to obtain an improved machine learning algorithm as a whole (36). XGBoost has been shown to perform exceptionally well in a variety of tasks in the areas of bioinformatics and medicine, such as the lysine glycation sites prediction for Homo sapiens (37), the chronic kidney disease diagnosis (38), and the risk prediction of incident diabetes (39). Also, random forest classifier is an ensemble algorithm, which combines multiple decorrelated decision tree prediction variables based on each subset of data samples (40). In general, random forest shows better performance in disease diagnosis than many single classifiers (41).

## Conclusion

In this study, we proposed a UDIPM embedding the disease ontology structure to identify and predict a UD from EMRs to assist better coding integration of diagnosis in the ICU. We discussed many critical issues, including a formal representation of multi-type patient information, symptom feature extraction from an unstructured discharge report, ICD ontology structure reconstruction for semantic relation embedding, multi-level set similarity measure for generating a patient similarity matrix, number of cluster selections using AP clustering, stability of the extracted TDC and TDCCoP from each cluster, optimal split line determination for identifying a UD based on visual analysis and the CCoM of LCoP, feature fusion and selection using the IG-based method, and the performance evaluation of UD prediction using five classifiers. We verified the proposed UDIPM on 4,418 sepsis patients in the ICU extracted from the MIMIC-III database. The results showed that the highest stability cluster number and largest range of TDCs were 2 and 400–800, respectively, the UD of cluster 2 was diseases of the genitourinary system (580–629, order: 1), septicemia (38.9, order: 2), and diseases of the respiratory system (460–519, order: 3), and the best AUC and Acc, Pre, Rec, and F of the UD prediction were 0.866, 0.795, 0.803, 0.795, and 0.794, respectively, which were better than those of other fusion methods from the overall view of SS and prediction performance.

## Study Limitations

The proposed UDIPM can identify and predict a UD from EMRs; however, there remain several topics for future work. First, the order of diagnosis codes should be considered in the patient similarity measure by way of different weights because of the importance of primary diseases. Then some state-of-the-art feature selection and classification models should be implemented to improve the prediction accuracies of the UD. Additionally, we hope to make progress on many of the valuable suggestions made by clinicians regarding our implemented method and experimental results.

## Data Availability Statement

Publicly available datasets were analyzed in this study. This data can be found at: https://mimic.mit.edu/docs/iii/tables/.

## Author Contributions

JC, CG, and SD conceived and designed the study and revised the manuscript. JC and ML carried out the experiments and drafted the manuscript. All the authors read and approved the manuscript.

## Funding

This work was supported by the National Natural Science Foundation of China (Grant Nos. 71771034, 72101236, and 71421001), the Scientific and Technological Innovation Foundation of Dalian (Grant No. 2018J11CY009), the Henan Province Youth Talent Promotion Project (Grant No. 2021HYTP052), the Henan Province Medical Science and Technology Research Plan (Grant No. LHGJ20200279), and the Henan Province Key Scientific Research Projects of Universities (Grant No. 21A320035).

## Conflict of Interest

The authors declare that the research was conducted in the absence of any commercial or financial relationships that could be construed as a potential conflict of interest.

## Publisher's Note

All claims expressed in this article are solely those of the authors and do not necessarily represent those of their affiliated organizations, or those of the publisher, the editors and the reviewers. Any product that may be evaluated in this article, or claim that may be made by its manufacturer, is not guaranteed or endorsed by the publisher.

## Figures and Tables

**Table:** 

*dc_i_*	*i*-th diagnosis code
*Ord*(*dc_i_*)	Order of *dc_i_*
*LCA*(*dc_i_, dc_j_*)	Least common ancestor of *dc_i_* and *dc_j_*
*s*(*dc_i_, dc_j_*)	Similarity of diagnosis code *dc_i_* and *dc_j_*
*S*(*D_i_*′, *D_j_*′)	Similarity of diagnostic information of patients *i* and *j*
**S**	Patient similarity matrix based on diagnostic information
*p_c_*	*p* coefficient to control the input exemplar preferences
*K*	Number of clusters
*C_k_*	*k*-th cluster, *k* = 1, 2,…, *K*
*E*(*C_k_*)	Exemplar of cluster *C_k_*
*Core_k_, |Core_k_|*	Core zone and the number of patients in *C_k_*
*Prob_k_* (*dc_h_*)	Occurrence probability of the diagnosis code *dc_h_* in *C_k_*
*AOrd_k_* (*Tdc_h_*)	Average order of the typical diagnosis code *dc_h_* in *C_k_*
*Ord'* (*Tdc_h_*)	New order of the typical diagnosis code *dc_h_*
*TDCCoP_k_*	*k*-th typical diagnosis code co-occurrence pattern
*LCoP_k_*	*k*-th least common ancestor co-occurrence pattern
**CCoM** *_k_*	Conditional co-occurrence matrix for all diseases in *TDCoP_k_*
*UD_k_*	*k*-th unifying diagnosis
*IG*(*x_i_*)	Information gain of feature *x_i_*
*CVError_Z_*	Average error using *Z*-fold cross-validation

## Abbreviations

EMR: Electronic medical record
UDIPM: Unifying diagnosis identification and prediction method
CDSS: Clinical decision support system
ICU: Intensive care unit
IC: Information content
LCA: Least common ancestor
AP: Affinity propagation
SS: Sum of similarities
TDC: Typical diagnosis code
LCoP: LCA co-occurrence pattern
AOrd: Average order
TDCCoP: Typical diagnosis code co-occurrence pattern
CCoM: Conditional co-occurrence matrix
UD: Unifying diagnosis
Hadm-id: Hospital admission identifier
FM: Fusion method.
